# Complete Genome Sequence of *Enterobacter cloacae* B2-DHA, a Chromium-Resistant Bacterium

**DOI:** 10.1128/genomeA.00483-16

**Published:** 2016-06-02

**Authors:** Aminur Rahman, Noor Nahar, Björn Olsson, Abul Mandal

**Affiliations:** Systems Biology Research Center, School of Bioscience, University of Skövde, Skövde, Sweden

## Abstract

Previously, we reported a chromium-resistant bacterium, *Enterobacter cloacae* B2-DHA, isolated from the landfills of tannery industries in Bangladesh. Here, we investigated its genetic composition using massively parallel sequencing and comparative analysis with other known *Enterobacter* genomes. Assembly of the sequencing reads revealed a genome of ~4.21 Mb in size.

## GENOME ANNOUNCEMENT

The chromium-resistant strain B2-DHA was isolated from the landfills of leather manufacturing tannery industries in the Hazaribagh area, in very close vicinity of the capital city Dhaka, Bangladesh, where the tannery wastes have been disposed for many years (1). Sequencing of the genomic DNA of B2-DHA was performed by an Illumina sequencer HiSeq-2500 PE106 (106-bp paired-end) with a single sequencing index. Read quality checks were performed with FastQC (2) version 0.10.1. Adapter and quality trimming on raw reads were conducted with Cutadapt (3). k-mer error correction was performed on the adapter-free reads using Quake version 0.3.5 (4). Properly paired reads were extracted from the corrected read pool, and the remaining singleton reads were listed as single-end reads. Both corrected paired-end and single-end reads are used in the subsequent *de novo* assembly. SOAP*denovo* (5) version 2.04 was utilized to perform *de novo* assembly optimization with the error-corrected reads. A wide range of k-mers (29 to 99) was tried to identify the scaffold sequences with the maximal *N*_50_. The largest *N*_50_, 492,970 bp, was produced at the k-mer 97.

A total of 1,756,877,072 bases and 16,574,312 pairs of reads were generated by Illumina deep sequencing. Analysis of the raw reads with FastQC showed that the average per-base Phred score was ≥36 for all positions, and the mean per-sequence Phred score was 36. The overall G+C content was 55%. After quality trimming, error correction, and removal of the TruSeq adaptor sequence, 15,708,650 read pairs (94.78%) and 331,106 single-end sequences remained for further analysis. The set of scaffold sequences with maximal *N*_50_ (492,970 bp) was produced at a k-mer of 97. The corresponding scaffold sequences were subjected to gap closure using the corrected paired-end reads, and the resulting scaffolds (≥24,300 bp) were defined as the final assembly. The final assembly was 4,218,945 bp and consisted of 13 scaffolds ranging from 72,208 bp to 777,700 bp.

The assembled genome sequence was annotated with the RAST (6) and Blast2GO (7) pipelines. ARAGORN (8) version 1.2.36 was used to predict tRNA genes. Prediction of tRNA-, rRNA- and protein-coding genes was performed based on RAST-predicted RNA genes. RAST resulted in 22 rRNA genes, including four long subunit (LSU), 4 short subunit (SSU), eight 16S, and six 23S genes. GeneMark (9) and FGenesB (10) algorithms were applied, yielding 3,764 and 3,955 genes, respectively. A total of 3,955 protein-coding genes were predicted using FGenesB, of which 3,159 could be annotated by the Blast2GO pipeline. The functional annotation by RAST and Blast2GO indicated that B2-DHA contains many genes that are responsive to binding metal ions, like chromium, cobalt, copper, iron, arsenic, nickel, manganese, zinc, and potassium. For functional annotation, all protein-coding sequences resulting from GeneMark were used by Blast2GO. Based on the phylogenetic trees inferred by using the neighbor-joining method (11) presented in the MEGA6 software (12), B2-DHA resembles *Enterobacter cloacae* KMBC1 and *E. cloacae* EC7. Previously, we have also reported a very high arsenic resistant bacterium *Lysinibacillus sphaericus* B1-CDA which harbors genes responsive to several metals such as arsenic, nickel, cadmium, iron, manganese, chromium, cadmium, lead, cobalt, zinc, silver and mercury (13–14).

In summary, the strain B2-DHA harbors several metal-responsive genes that might be utilized in the bioremediation of chromium and other toxic metals in polluted environments.

### Nucleotide sequence accession numbers.

The genome sequence of B2-DHA strain has been registered in GenBank under accession no. LFJA00000000. The version described in this paper is the first version, LFJA00000000.1.

## References

[1] Rahman A, Nahar N, Nawani NN, Jass J, Hossain K, Saud ZA, Saha AK, Ghosh S, Olsson B, Mandal A 2015 Bioremediation of hexavalent chromium (VI) by a soil- borne bacterium, *Enterobacter cloacae* B2-DHA. J Environ Sci Health, Part A 50:1136–1147. doi:10.1080/10934529.2015.1047670. 26191988

[2] Andrews S 2010 FastQC: a quality control tool for high throughput sequence data. http://www.bioinformatics.babraham.ac.uk/projects/fastqc.

[3] Martin M 2011 Cutadapt removes adapter sequences from high-throughput sequencing reads. EMBnet.J 17:10–12. doi:10.14806/ej.17.1.200.

[4] Kelley DR, Schatz MC, Salzberg SL 2010 Quake: quality-aware detection and correction of sequencing errors. Genome Biol 11:R116. doi:10.1186/gb-2010-11-11-r116. 21114842 PMC3156955

[5] Luo R, Liu B, Xie Y, Li Z, Huang W, Yuan J, He G, Chen Y, Pan Q, Liu Y, Tang J, Wu G, Zhang H, Shi Y, Liu Y, Yu C, Wang B, Lu Y, Han C, Cheung DW, Yiu SM, Peng S, Xiaoqian Z, Liu G, Liao X, Li Y, Yang H, Wang J, Lam TW, Wang J 2012 SOAP*denovo*2: an empirically improved memory-efficient short-read *de novo* assembler. GigaScience 1:18. doi:10.1186/2047-217X-1-18. 23587118 PMC3626529

[6] Aziz RK, Bartels D, Best AA, DeJongh M, Disz T, Edwards RA, Formsma K, Gerdes S, Glass EM, Kubal M, Meyer F, Olsen GJ, Olson R, Osterman AL, Overbeek RA, McNeil LK, Paarmann D, Paczian T, Parrello B, Pusch GD, Reich C, Stevens R, Vassieva O, Vonstein V, Wilke A, Zagnitko O 2008 The RAST server: Rapid Annotations using Subsystems Technology. BMC Genomics 9:75. doi:10.1186/1471-2164-9-75. 18261238 PMC2265698

[7] Götz S, García-Gómez JM, Terol J, Williams TD, Nagaraj SH, Nueda MJ, Robles M, Talón M, Dopazo J, Conesa A 2008 High-throughput functional annotation and data mining with the Blast2GO suite. Nucleic Acids Res 36:3420–3435. doi:10.1093/nar/gkn176. 18445632 PMC2425479

[8] Laslett D, Canback B 2004 ARAGORN, a program for the detection of transfer RNA and transfer-messenger RNA genes in nucleotide sequences. Nucleic Acids Res 32:11–16. doi:10.1093/nar/gkh152. 14704338 PMC373265

[9] Borodovsky M, McIninch J 1993 GENMARK: parallel gene recognition for both DNA strands. Comput Chem 17:123–133. doi:10.1016/0097-8485(93)85004-V.

[10] Salamov AA, Solovyev VV 2000 *Ab initio* gene finding in *Drosophila* genomic DNA. Genome Res 10:516–522. doi:10.1101/gr.10.4.516. 10779491 PMC310882

[11] Saitou N, Nei M 1987 The neighbor-joining method: a new method for reconstructing phylogenetic trees. Mol Biol Evol 4:406–425. 3447015 10.1093/oxfordjournals.molbev.a040454

[12] Tamura K, Stecher G, Peterson D, Filipski A, Kumar S 2013 MEGA6: Molecular Evolutionary Genetics Analysis version 6.0. Mol Biol Evol 30:2725–2729. doi:10.1093/molbev/mst197. 24132122 PMC3840312

[13] Rahman A, Nahar N, Nawani NN, Jass J, Ghosh S, Olsson B, Mandal A 2015 Comparative genome analysis of *Lysinibacillus* B1-CDA, a bacterium that accumulates arsenics. Genomics 106:384–392. doi:10.1016/j.ygeno.2015.09.006. 26387925

[14] Rahman A, Nahar N, Jass J, Olsson B, Mandal A 2016 Complete genome sequence of *Lysinibacillus sphaericus* B1-CDA, a bacterium that accumulates arsenic. Genome Announce 4(1):e00999-15. doi:10.1128/genomeA.00999-15. PMC4722251 26798084

